# C5L2 modulates BDNF production in human dental pulp stem cells via p38α pathway

**DOI:** 10.1038/s41598-022-27320-6

**Published:** 2023-01-02

**Authors:** Muhammad Irfan, Seung Chung

**Affiliations:** Department of Oral Biology, College of Dentistry, University of Illinois Chicago, 801 S. Paulina St, Chicago, IL 60612 USA

**Keywords:** Stem cells, Mesenchymal stem cells

## Abstract

Tissue injury affects nerve fibers and triggers an immune response, leading to inflammation. The complement system gets activated during inflammatory conditions and has been reported to be involved in the regeneration process. We have demonstrated that the C5a receptor (C5aR) has crucial roles in regeneration and healing processes including nerve sprouting and hard tissue formation. Another C5a-like 2 receptor (C5AR2; C5L2) has been cloned which is still considered controversial due to limited studies. We previously established that C5L2 regulates brain-derived neurotrophic factor (BDNF) secretion in pulp fibroblasts. However, there is no study available on human dental pulp stem cells (DPSCs), especially in the inflammatory context. Stem cell therapy is an emerging technique to treat and prevent several diseases. DPSCs are a great option to be considered due to their great ability to differentiate into a variety of cells and secrete nerve regeneration factors. Here, we demonstrated that C5L2 modulates BDNF secretion in DPSCs. Our results stated that C5L2 silencing through siRNA could increase BDNF production, which could accelerate the nerve regeneration process. Moreover, stimulation with lipopolysaccharide (LPS) enhanced BDNF production in C5L2 silenced DPSCs. Finally, we quantified BDNF secretion in supernatant and cell lysates using ELISA. Our results showed enhanced BDNF production in C5L2 silenced DPSCs and hampered by the p38^MAPK^α inhibitor. Taken together, our data reveal that C5L2 modulates BDNF production in DPSCs via the p38^MAPK^α pathway.

Stem cell therapy is one of the recent advances in regenerative medicine. Cells that can differentiate into neurons or secrete neurotrophic factors to promote nerve regeneration, are gaining attention^1^. Dental pulp stem cells (DPSCs) are derived from neural crest which could give rise to glia and/or neurons due to their similarities to the neuronal cells and strong expression of neuronal markers, suggesting that DPSCs could actively adapt to neuronal environment^2–4^. DPSCs are known for their neurogenic and regenerative ability^2^. DPSCs show better neuroregeneration and protective ability than bone marrow-derived mesenchymal cells (BM-MSCs) and are easy to harvest with minimally invasive procedures, making them a better choice for neural regeneration^5,6^.

Nerve repair and regeneration have been a major concern in several disease conditions and surgical interventions which requires progenitor cells to passage and differentiate for repairing nerve portions. Cells that could provide an environment for matrix protein and secretion of neurotropic factors e.g., nerve growth factor (NGF) and brain-derived neurotrophic factor (BDNF), etc., facilitate progenitor cells proliferation and differentiation^7^. The short life of these neurotrophic factors is of utmost apprehension, e.g., the human BDNF recombinant protein has a short life of less than 10 min which limits its efficiency^8^. Therefore, a constant source is required to achieve the maximum neural repair.

The complement system is a part of innate immunity and gets activated during inflammation which is also known to facilitate tissue regeneration ^9,10^. Complement C5a receptor has been studied for its beneficial effects on several tissues regeneration^11–13^. We recently studied the role of complement C5a receptor in tooth regeneration^14,15^ and neurite outgrowth^16^ including C5aR-mediated enhanced BDNF secretion^17^. Another C5a-like receptor 2 (C5AR2; C5L2) has been cloned which is still a controversial receptor and received much less attention due to limited available studies, also it is known to work opposite to the C5aR^18^. Previously, we have studied the role of C5L2 in odontoblastic differentiation and nerve growth factors secretions using pulp fibroblasts and showed that C5L2 siRNA silencing could enhance the regenerative ability of pulp fibroblasts ^19–21^. The aim of this study was to evaluate the effect of C5L2-mediated BDNF production in DPSCs under LPS-induced inflammation.

## Results

### C5L2 siRNA silencing in STRO-1 positive DPSCs

**Figure 1:**
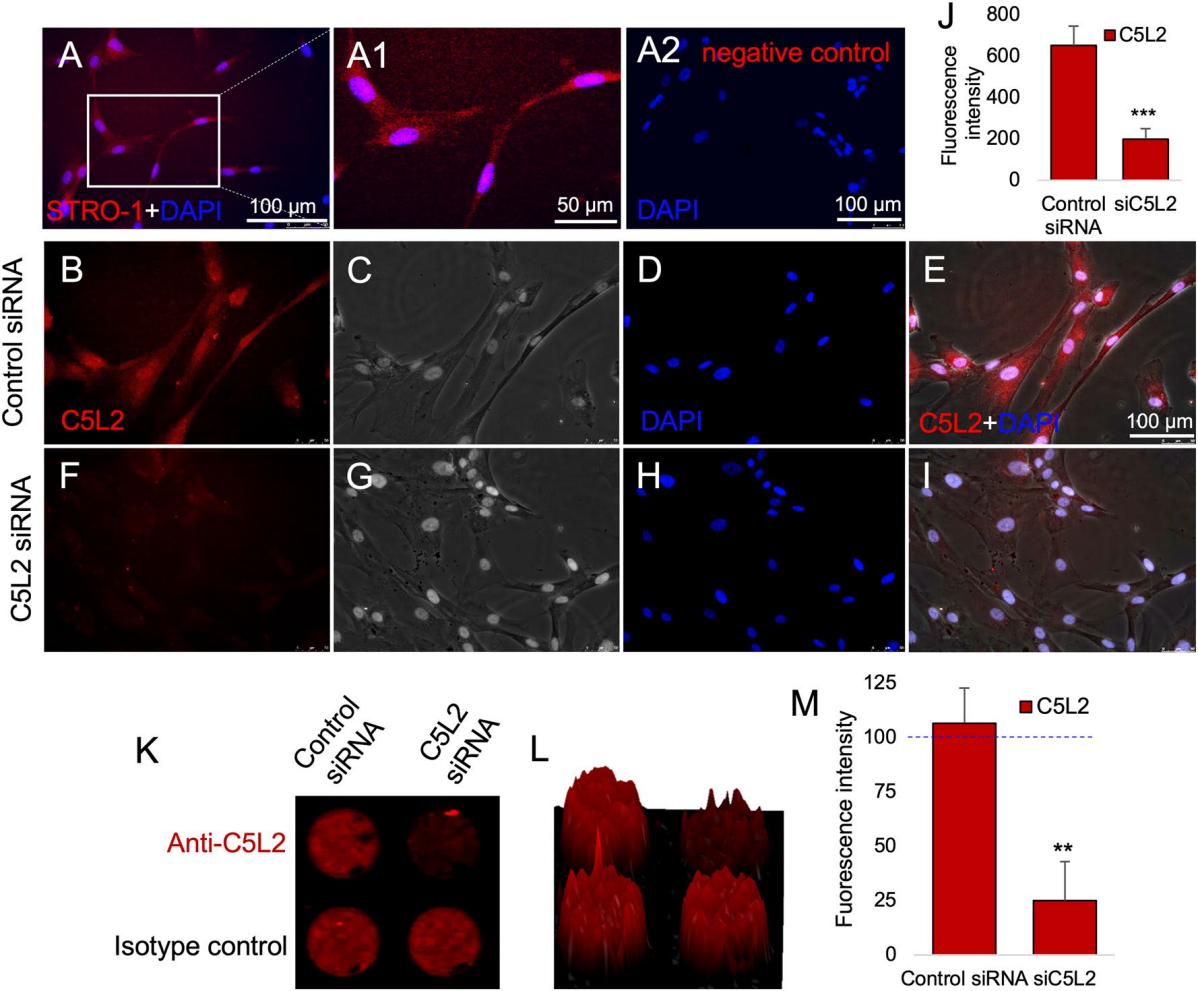
C5L2 siRNA treatment knocked down the C5L2 in DPSCs. (**A**-**A1**) Commercial DPSCs were further confirmed with mesenchymal stem cell marker STRO-1. **(A2)** negative control for antibody. **(B-I)** To confirm siRNA silencing efficiency, immunostaining was implemented on DPSCs cultured and transfected with control siRNA or C5L2 siRNA. Differential interference contrast (DIC) images indicate the absence of structural and morphological disparity between the 2 different treatments (**C**, **G**). Nuclei were counterstained with DAPI (**D**, **H**). C5L2 is expressed in all DPSCs transfected with control siRNA (**E**), whereas its expression is drastically reduced in DPSCs transfected with C5L2 siRNA (**I**). C5L2 staining and differential interference contrast (DIC) merge images show C5L2 mainly localized in the intracellular compartment and confirm C5L2 siRNA silencing (**E**, **I**). **(J)** Bar graph shows a significant reduction in integrated fluorescence intensity among DPSCs treated with control siRNA or C5L2 siRNA, and clearly indicates C5L2 silencing in C5L2 siRNA transfected DPSCs (*** *p* < 0.001 vs control). To further validate C5L2 silencing, an in-cell western assay was performed **(K-M)**. C5L2 siRNA-treated well clearly indicated the C5L2 silencing compared with control siRNA treated well (**K**). The image was analyzed to quantify the integrated fluorescence intensity using a 2.5D model which further attested the C5L2 silencing in C5L2 siRNA transfected cells (**L**). (**M**) Bar graph shows a significant reduction in integrated fluorescence density in C5L2 siRNA treated well vs control siRNA (** *p* < 0.01 vs control).

Commercially available DPSCs were further confirmed by mesenchymal stem cell marker, i.e., STRO-1 (Fig. 1A,A1,A2 as negative control). C5L2 siRNA silencing was performed using a siRNA reagent system (Fig. 1B–J). Figure 1B shows the presence of C5L2 distributed in the DPSCs while a clear inhibition in the expression can be seen in Fig. 1F, after treating the cells with C5L2 siRNA. The bar graph indicates the significant inhibition and silencing of C5L2 (198.2 ± 50.9, *p* < 0.001 vs control 652 ± 92) in DPSCs (Fig. 1J). C5L2 siRNA silencing was further confirmed by in-cell western assay which shows clear inhibition compared to the control (Fig. K). The integrated fluorescence intensity was quantified using a 2.5D model (Fig. 1L) and bar graph (Fig. 1M) representation. In Fig. 1M, higher expression of C5L2 in the control group and least in the C5L2 siRNA treated group is obvious (25 ± 18, *p* < 0.001 vs control 106.6 ± 16).

### C5L2 silencing enhances BDNF production in DPSCs

**Figure 2:**
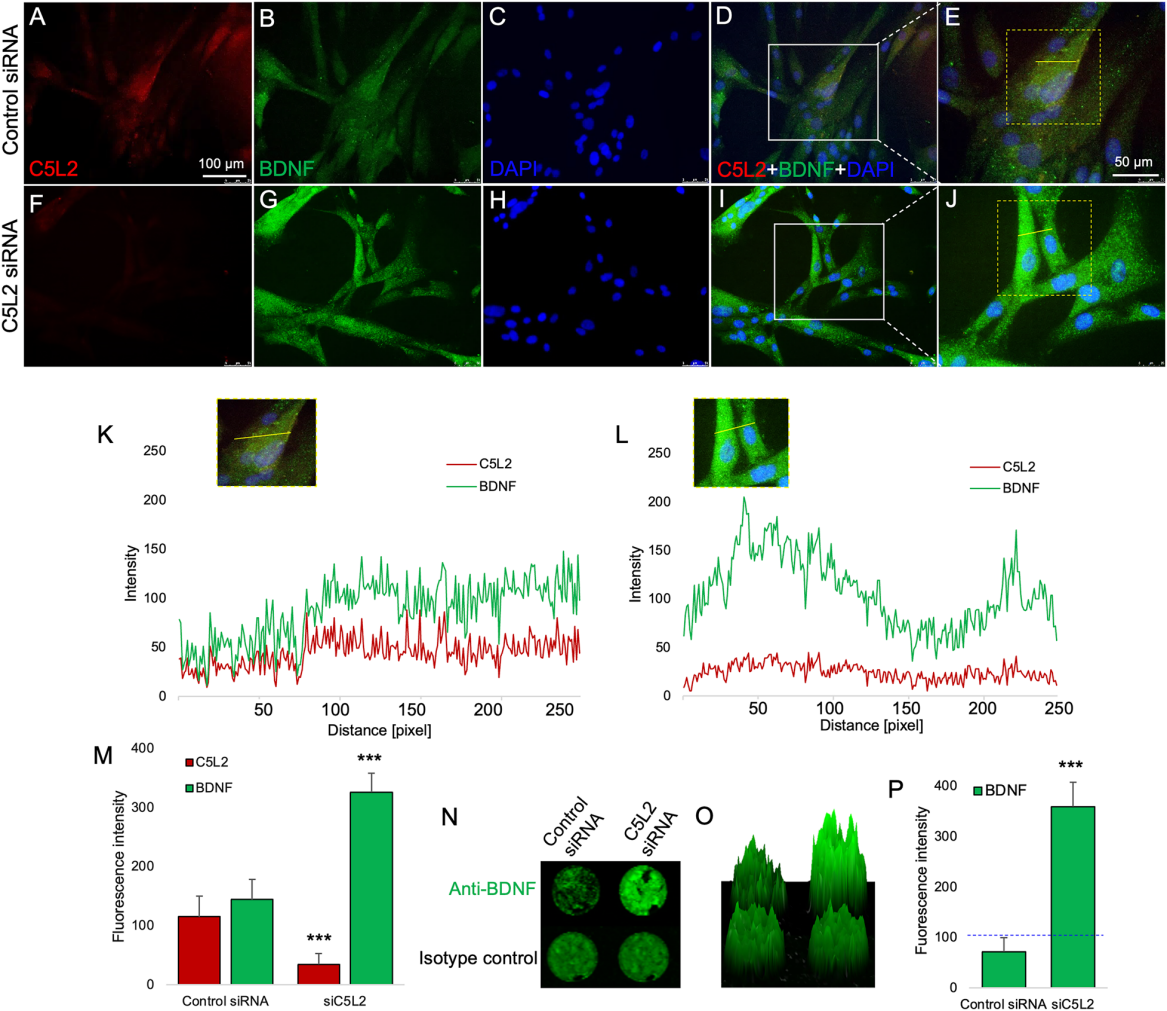
Effect of C5L2 silencing on BDNF expression in DPSCs. (**A**-**J**) Cells were cultured and transfected with control siRNA or C5L2 siRNA and immunofluorescence double staining was used to analyze the expression of C5L2 and BDNF among DPSCs. DAPI was used to counterstain nuclei. Remarkably, C5L2 silencing enhanced BDNF expression in DPSCs (**F**-**J**) compared with control (**A-E**). (**K-L**) Enlarged Fig. E and J were analyzed showing co-localization of C5L2 and BDNF in control siRNA-treated cells (**K**) and enhanced BDNF expression with reduced C5L2 expression (**L**) indicating that C5L2 silencing enhances BDNF expression in DPSCs. (M) Bar graph shows increased fluorescence intensity of BDNF among siC5L2 treated cells (**F-J**) compared with control (**A-E**). (**N-P**) Similarly, the in-cell western assay showed increased immunofluorescence of BDNF in C5L2 silenced well compared to control. (**O**) 2.5D model depicting similar results displaying a high peak of BDNF among C5L2 silenced cells. (**P**) Bar graph shows significant increment in BDNF expression in C5L2 silenced DPSCs (***p < 0.001 vs control).

BDNF secretion facilitates neuronal growth. It is fairly predictable that BDNF secretion from DPSCs could help rising neuronal growth via the paracrine effect^22^. Therefore, we investigated the effect of C5L2 inhibition on increased production of BDNF. Our results reveal that C5L2 siRNA treated cells express significantly higher BDNF than the control group Fig. 2A–J. The co-localization analysis further confirmed the difference in C5L2 and BDNF expression. Control cells show co-localization of both BDNF and C5L2 expression with green and red signal peaks (Fig. 2K). Correspondingly, higher BDNF expression in C5L2 siRNA treated cells with five times higher peaks of green signal compared to red signal for C5L2 expression (Fig. 2L). Figure 2M shows significantly increased BDNF fluorescence intensity in C5L2 silenced cells (326.3 ± 32, *p* < 0.001) compared with control siRNA treatment group (144 ± 33.9). BDNF expression was further analyzed by in-cell western assay which provided further evidence that inhibiting C5L2 could enhance BDNF expression (Fig. 2N). Similarly, 2.5D model shows a clear difference with higher BDNF expression in C5L2 siRNA treated cells with a higher intensity peak compared to the control well (Fig. 2O). In Fig. 2P, bar graph shows a five times higher integrated fluorescence density of BDNF expression in C5L2 siRNA treated cells (359.3 ± 48.2, *p* < 0.001) compared to control siRNA treated cells (71.66 ± 27.5).

### LPS potentiate BDNF production in C5L2 silenced DPSCs

**Figure 3:**
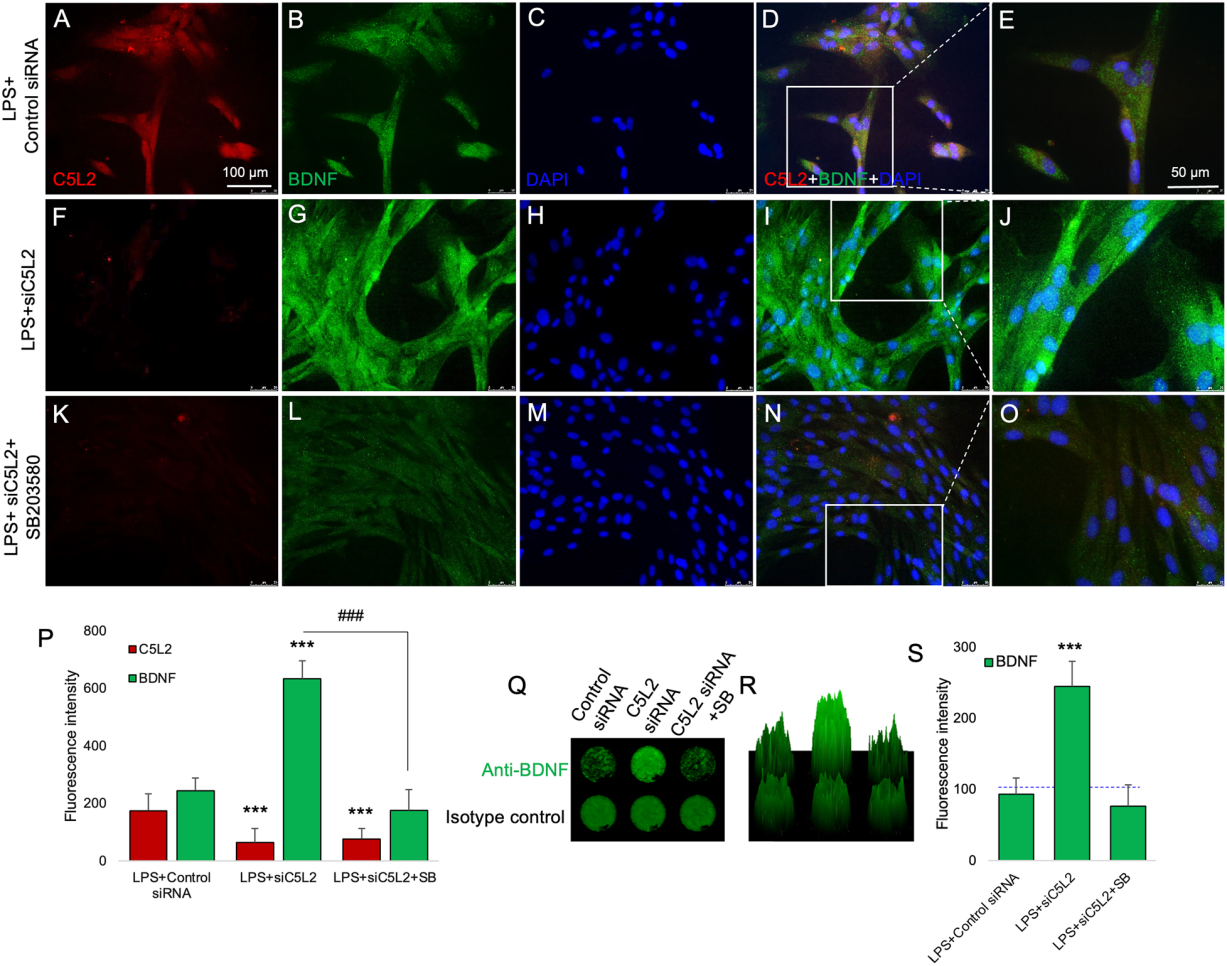
Effect of LPS and p38^MAPK^ α inhibitor on BDNF secretion in C5L2 silenced DPSCs. (**A-O)** Cells were cultured and transfected with control siRNA, or C5L2 siRNA or treated with p38^MAPK^ α inhibitor (SB203580; SB), stimulated with LPS. Immunofluorescence double staining was used to analyze C5L2 and BDNF expression under LPS stimulation. LPS stimulation enhanced BDNF expression in C5L2 silenced DPSCs under LPS stimulation (**F**-**J**) compared with control (**A**-**E**). SB203580 treatment inhibited the BDNF expression in C5L2 silenced DPSCs, indicating p38α involvement in C5L2-mediated BDNF expression (**K**–**O**). Enlarged figures (**E**, **J**, and **O**) show co-localization of C5L2 and BDNF, and C5L2 silenced cells stimulated with LPS (**J**) clearly indicated the increased expression of BDNF. (**P**) The bar graph shows a significant increment in BDNF expression with reduced C5L2 expression while SB203580 treatment obliterated the effects (***p < 0.001 vs control). (**Q**-**S**) In-cell western assay further confirmed the immunofluorescence staining results. C5L2 silencing increased the BDNF expression while SB203580 treatment inhibited the BDNF expression in LPS-stimulated C5L2 silenced cells, indicating the role of p38α in C5L2 mediated BDNF expressing DPSCs. 2.5D model (**R**) and the bar graph (**S**) shows a significant increment in BDNF expression compared with control while the effect reversed in SB203580 treated cells (***p < 0.001 vs control).

It is known that inflammation helps tissue regeneration. Our results show that controlled or choreographed inflammation might enhance BDNF (Fig. 3A–J). It can be seen in Fig. 3F,G that C5L2 siRNA-treated cells show higher BDNF expression compared to the control. In Fig. 3P, the bar graph also shows that C5L2 siRNA treatment under LPS stimulation significantly enhanced the BDNF expression (633.2 ± 62, *p* < 0.001) compared to siC5L2 alone (326.3 ± 32; Fig. 2M) or LPS alone (245 ± 43.1) which is comparable with control siRNA treated cells without LPS, *i.e.*, 144 ± 33.9 (Fig. 2M). And a similar pattern of increased BDNF expression in C5L2 silenced cells was observed through in-cell western technique (Fig. 3Q–S). These data show that inflammation could modulate DPSCs functions via increased BDNF production and decreased C5L2 expression.

### p38α involved in C5L2 mediated BDNF production

Mitogen-activated protein kinase is known to be involved in several important biological functions and modify the microcellular pathways. It is also known that p38^MAPK^ could interfere with differentiating DPSCs^23^. We treated differentiating DPSCs with a p38α inhibitor and interestingly the BDNF secretion in C5L2 silenced cells was diminished (Fig. 3K–O). Figure 3P shows the significant reduction in BDNF production with p38α inhibitor treatment (176 ± 62.5, *p* < 0.001 compared with LPS-stimulated C5L2 silenced cells). Similar observations were made with in-cell western technique (Fig. 3Q), testified by 2.5D model (Fig. 3R). The data were quantified as integrated fluorescence intensity (Fig. 3S) showing a significant increment in the BDNF expression (244.66 ± 35.5, *p* < 0.001) compared to control.

**Figure 4:**
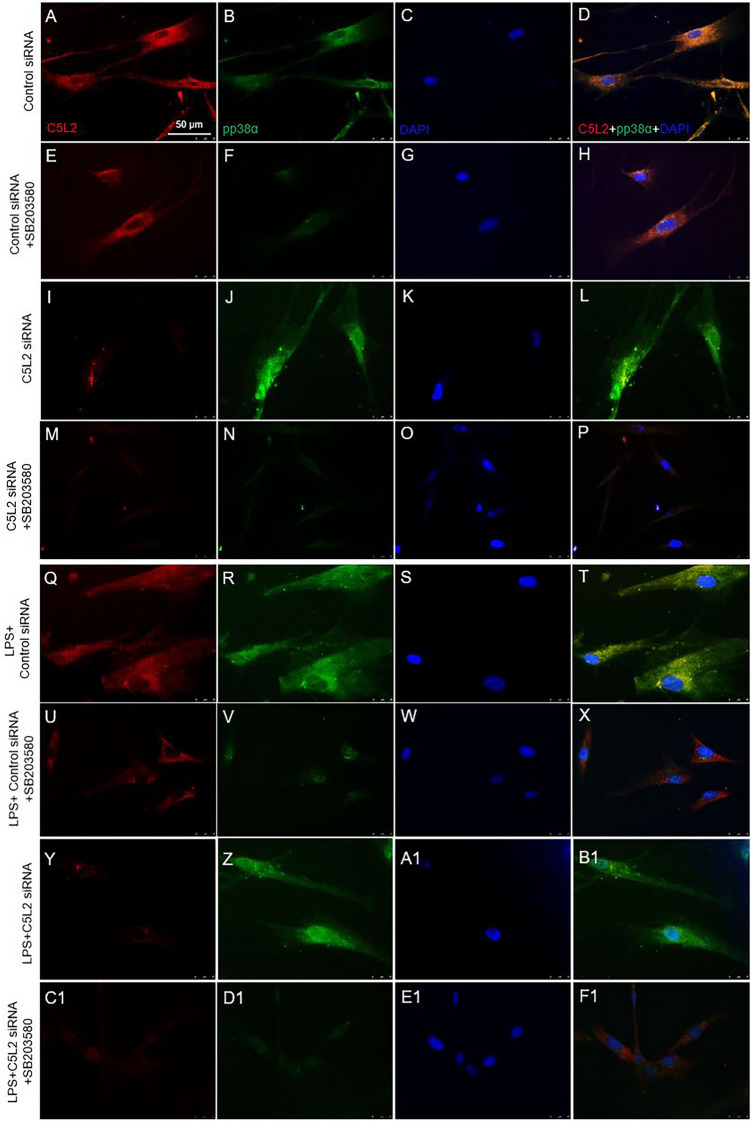

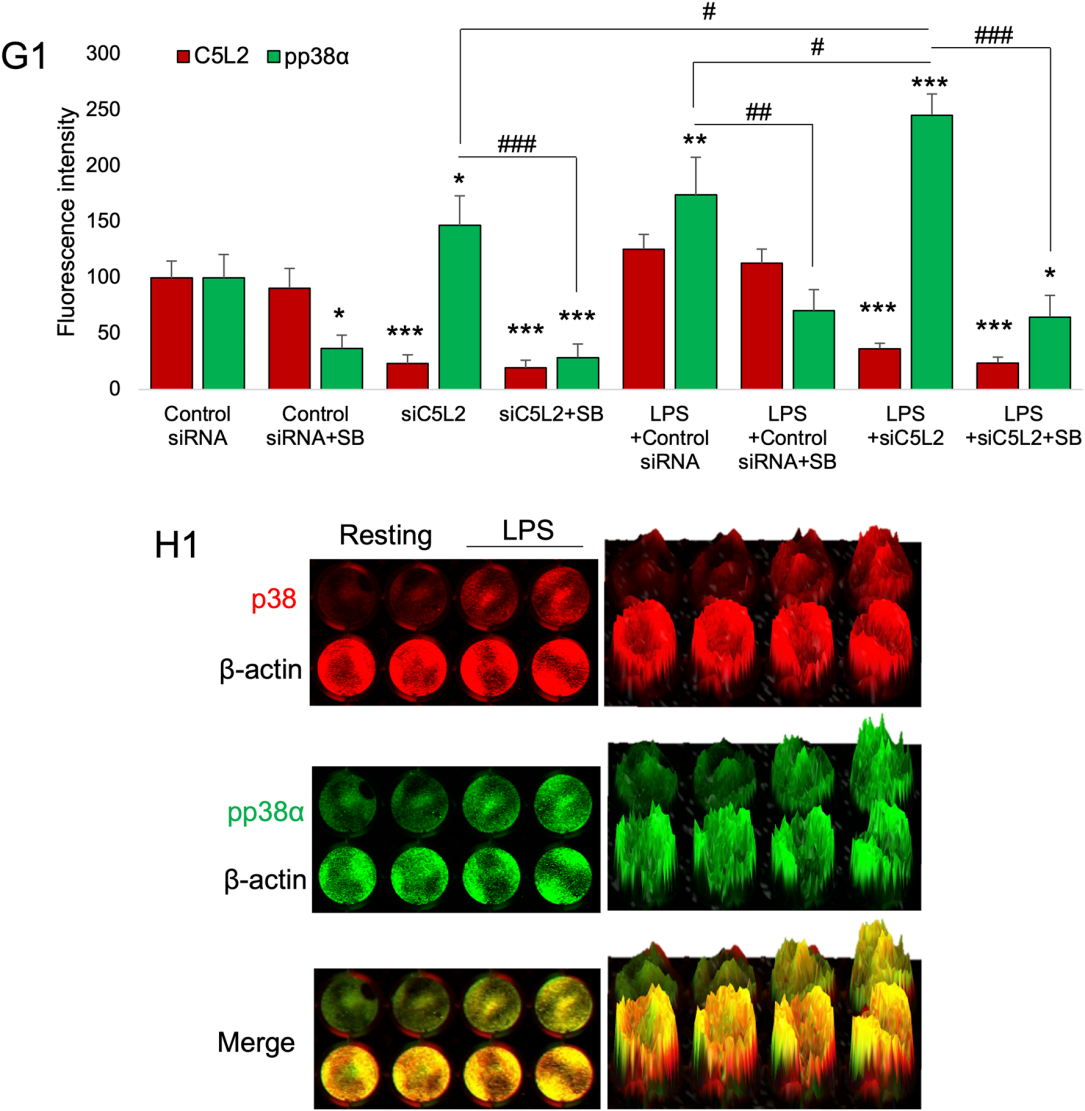
Effects of LPS on C5L2 mediated p38α phosphorylation in DPSCs. (**A**-**F1**) Cells were cultured and treated with control siRNA (**A**-**D**), Control siRNA + SB203580 (E–H), C5L2 siRNA (I-L), or C5L2 siRNA + SB203580 (**M**-**P**), and/or stimulated with LPS (**Q**-**F1**). Immunofluorescence double staining was used to analyze C5L2 and pp38α with or without LPS stimulation. C5L2 silencing enhanced phosphorylation of p38α in DPSCs with or without LPS (**I**-**L**; **Y**-**B1**) compared with cells treated with control siRNA alone or with LPS (**A**-**D**; **Q**-**T**). Interestingly, SB203580 treatment with C5L2 siRNA reversed the effects and reduced the phosphorylation of p38α with or without LPS stimulation (M-P; C1-F1). (**G1**) The bar graph shows a significant increase in p38α phosphorylation while SB203580 treatment remarkably reduced it. LPS stimulation enhanced the p38α phosphorylation and again SB203580 treatment reversed its effects (*p < 0.05, **p < 0.01, and ***p < 0.001 vs control; #*p* < 0.05, ##*p* < 0.01, and ###*p* < 0.001 vs respective line-indicated group). (**H1**) The in-cell western assay was performed to access p38 and p38α phosphorylation with or without LPS stimulation. It was observed that LPS stimulation significantly increase phosphorylation of p38 and p38α which further testify the above-mentioned phenomenon.

To further access the role of p38α^MAPK^, cells were treated with C5L2 siRNA and p38α inhibitor with or without LPS (Fig. 4A–F1). Interestingly, C5L2 siRNA silencing enhanced phosphorylation of p38α (Fig. 4I–L) compared to control, while p38α inhibitor treatment abolished this expression (Fig. 4M–P). Congruently, C5L2 and p38α phosphorylation showed a similar pattern in LPS-stimulated DPSCs (Fig. 4Q-F1). Bar graph shows that C5L2 silencing clearly increased the p38α phosphorylation in DPSCs (146.9 ± 26.2, *p* < 0.05) while p38α inhibitor (SB203580) abolished this expression (28.7 ± 11.9, *p* < 0.001) compared with control (Fig. 4 G1). LPS stimulation alone further enhanced p38α phosphorylation (174.1 ± 33.8, *p* < 0.01) and similar observation was with C5L2 silenced cells (245.33 ± 19.07, *p* < 0.001). The SB203580 treatment abolished these effects of C5L2 mediated p38α phosphorylation even under LPS stimulation (64.8 ± 19.5, *p* < 0.001) compared with control. Figure 4 H1 shows in-cell western assay results and we analyzed the data using 2.5D model, which states that p38, and pp38α expression increased in LPS-stimulated DPSCs compared with control, and our results showed that inhibiting p38α could interfere with C5L2 expression and BDNF production. These data show that p38α could interact with C5L2 downstream signaling and modulates BDNF secretion.

### BDNF quantification shows a linkage between C5L2 and p38α

**Figure 5:**
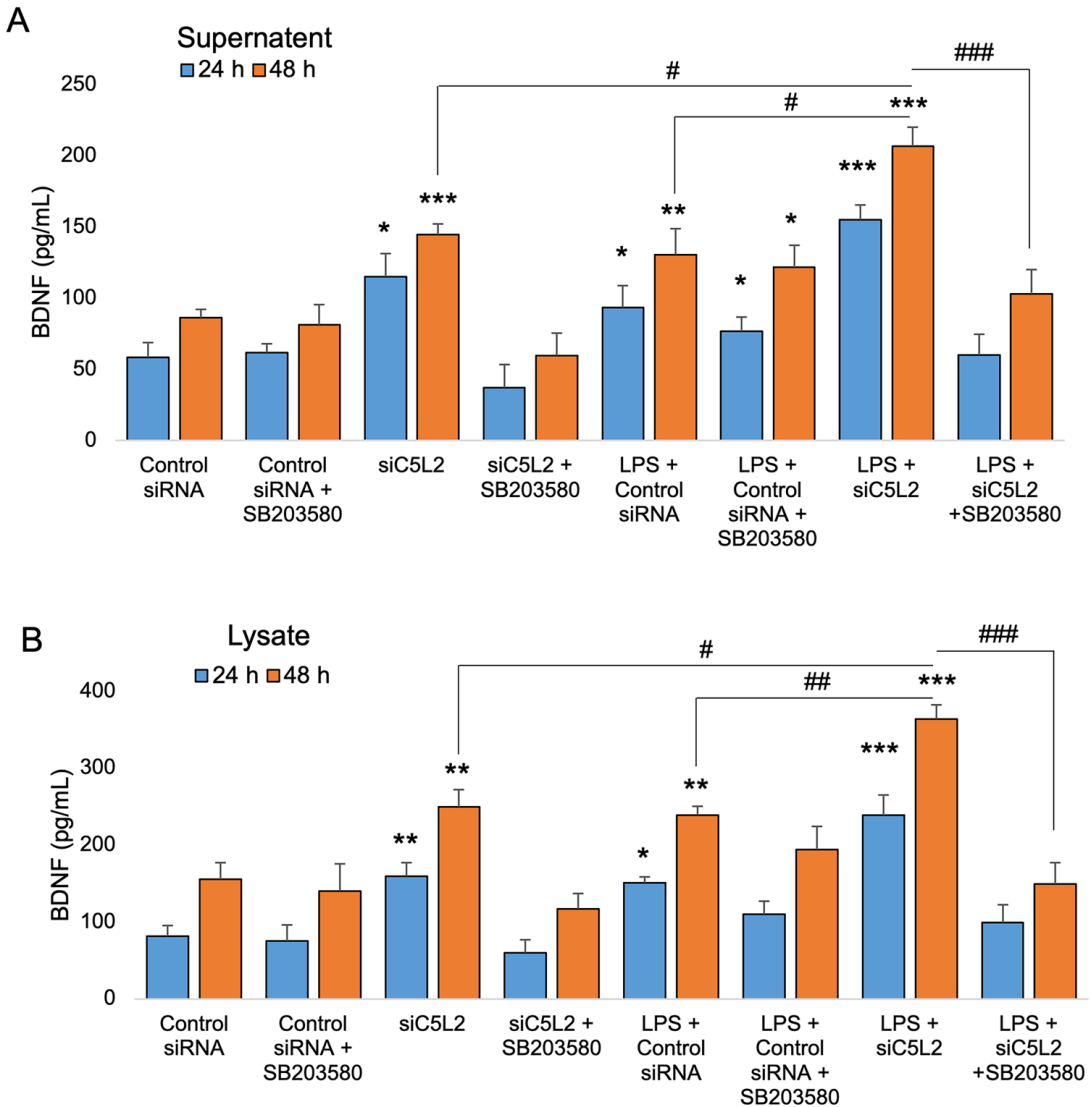
Effects of C5L2 mediated BDNF secretion in DPSCs in supernatant and cell lysates. (**A-B**) Cells were cultured and incubated with various treatments such as control siRNA, C5L2 siRNA, and/or SB203580 with or without LPS. Supernatant (conditioned media) or cell lysates were collected at 24 h and 48 h of treatments, and an ELISA was performed to quantify BDNF secretion in C5L2 mediated DPSCs according to the manufacturer’s protocol. (**A**) In supernatant, BDNF production was significantly increased in C5L2 silenced cells with or without LPS at both 24 h and 48 h. However, SB203580 treatment reversed the BDNF production. (**B**) Similar trend was observed in cell lysates with significant higher BDNF secretion as compared to cell supernatant (*p < 0.05, **p < 0.01, and ***p < 0.001 vs control; #*p* < 0.05, ##*p* < 0.01, and ###*p* < 0.001 vs respective line-indicated group).

To complement the immunocytochemistry and in-cell western data, we performed ELISA using both supernatant and cell lysate. Our results show the C5L2 mediated BDNF secretion in supernatant and lysate of DPSCs (Fig. 5). After 24 h of C5L2 silencing in the supernatant of DPSCs (Fig. 5A), the BDNF production was increased significantly (115 ± 16.1, *p* < 0.05) compared with resting control (58.3 ± 10.5). BDNF secretion was potentiated in LPS-stimulated DPSCs in C5L2 silenced cells (155 ± 10.5, *p* < 0.001) compared with siC5L2 alone (115 ± 16.1, *p* < 0.05) or LPS control (93.3 ± 15.5, *p* < 0.05).

After 24 h of silencing in the cell lysate (Fig. 5B), C5L2 siRNA-treated cells secreted a higher amount of BDNF (159.5 ± 17.5) compared with resting control cells (81.2 ± 14), while LPS stimulation significantly potentiated the BDNF production in LPS + C5L2 siRNA silenced DPSCs (238.6 ± 26, *p* < 0.01) compared to siC5L2 alone (159.5 ± 17.5) or LPS control (151.2 ± 7.5, *p* < 0.05).

To evaluate the role of p38α on C5L2 mediated BDNF production in DPSCs, SB203580 was used. Our results showed a significant reduction in BDNF secretion when treated with SB203580 in both supernatant (37.3 ± 16) and lysate (59.5 ± 17.5), and a similar pattern was observed in LPS-stimulated C5L2 siRNA and SB203580 treated groups of supernatant (60 ± 14.4) and lysate (99.5 ± 22). These results indicate that p38α regulates C5L2-mediated BDNF production in DPSCs.

## Discussion

The present study demonstrates the involvement of the complement system in stem cell-mediated neuroregeneration under inflammatory conditions. We explored the regenerative ability and the role of C5L2 on BDNF secretion in DPSCs. We evaluated the C5L2 siRNA-treated BDNF production in DPSCs with or without LPS and found that C5L2 silencing could enhance BDNF production via the p38α pathway suggesting that C5L2 negatively modulates BDNF production in DPSCs.

The complement system is one of the most critical components of innate immunity which can be activated during apoptosis, necrosis and bacterial or viral pathogenesis ^24–26^. Besides its role in immunity, it facilitates the process of regeneration e.g., it has been reported to be involved in the regeneration of bone, liver and cardiac tissues ^11–13^. Further, Bergmann et al., have reported the linkage between inflammation and tissue regeneration ^27^. The main effect of the complement system seems to be mediated by its active fragment C5a and its receptor C5aR. While another C5a-like receptor 2 (C5L2) received less attention and its biological functions remained inscrutable ^18,28^. Unlike most other G-protein coupled receptors (GPCRs), C5L2 is deficient in G-protein coupling, yet; C5L2 in an emerging functional receptor to be known for its roles in inflammation and regeneration ^29^. In our study, we used LPS-stimulated DPSCs and evaluated C5L2-mediated BDNF production which suggests a positive role in enhanced production of this indispensable neurotrophin.

Previously, we demonstrated the role of complement system in odontogenic differentiation and neural regeneration using pulp fibroblasts and suggested that complement receptors *i.e.*, C5aR and C5L2 facilitate dentin repair through DMP-1 expression and nerve fibers growth via increased BDNF secretion, respectively^19–21^. Recently, we studied C5aR-mediated BDNF production in DPSCs compared to bone marrow-derived mesenchymal stem cells (BM-MSCs) and concluded that DPSCs are a better choice for BDNF secretions than other stem cells when it comes to stem cell therapy in regenerative medicine^17^. In consistent with our study, Pagella et al. demonstrated that DPSCs are superior at enhancing nerve outgrowth via increased BDNF production compared to BM-MSCs ^22^. Another study showed that dental pulp-derived cells can differentiate into Schwann-like cells and secrete neurotrophins such as BDNF and NGF^30^. Several studies have established the capability of DPSCs to differentiate into neuronal cells and express neuronal markers ^2,31,32^. It is believed that the neural crest origin of DPSCs makes them an ideal choice for stem cell therapy, especially for neural regeneration. Considering several advantages of choosing DPSCs over other stem cells, we procured DPSCs for our study to evaluate the role of C5L2 in BDNF production.

Tissue regeneration following trauma and injury occurs in an inflammatory context. In this regard, we evaluated the effect of inflammation using LPS, which is one of the most potent inflammation inducers. We have previously established that lipoteichoic acid (LTA) stimulated dental pulp fibroblasts secrete higher levels of BDNF and enhanced axonal growth via C5aR modulation while C5aR antagonist abolished this potentiating effect and decreased axonal growth and length ^16^. Consistent with this, LPS treatment enhanced BDNF production in C5L2 silenced DPSCs suggesting a positive role of inflammation in DPSC-mediated tissue regeneration. We showed earlier that C5L2 silencing enhanced nerve outgrowth in dental pulp fibroblasts by increased BDNF secretion ^19,20^ and C5aR mediated nerve growth ^33^ suggesting a positive role of C5aR. Here, we studied the role of C5L2 siRNA silencing in BDNF secretion and our results showed increased BDNF production in C5L2 silenced DPSCs, which indicates a negative role of C5L2. Taken together, our results including our previous studies^19,20^ suggest that C5aR activity can be enhanced by C5L2 inhibition. The alternative approach of targeting C5L2 could provide an innovative therapeutic strategy, i.e., the possibility to enhance the positive action of C5aR in stem cell engineering and tissue regeneration by blocking the “inactive” C5L2 receptor.

The p38^MAPK^ molecule is highly expressed in the brain and is known to be involved in several biological activities including cell proliferation and differentiation ^34,35^. Recently, another study demonstrated that p38/BDNF coupled signaling mediates neurite outgrowth and neuronal survival ^36^. Engel et al., have discussed the hypothalamic neurogenesis p38^MAPK^ and BDNF-dependent mechanism ^37^. A previous study suggested one of the possible downstream pathways of C5a is p38, which showed its role in C5a-induced chemotactic cell migration^38^*.*

We also identified the role of p38 in the LPS-induced odontogenic differentiation of DPSCs (manuscript under review). LPS treatment increased the expression of p38, and activated p38, and treatment with the SB20358 abolished the LPS-induced DSPP and DMP-1 increase. These data suggest a crucial role of C5aR and its putative downstream target p38 in the LPS-induced odontogenic DPSCs differentiation. Our results are in accordance that depicts the possible involvement of p38^MAPK^ in C5L2-mediated BDNF secretion.

**Figure 6:**
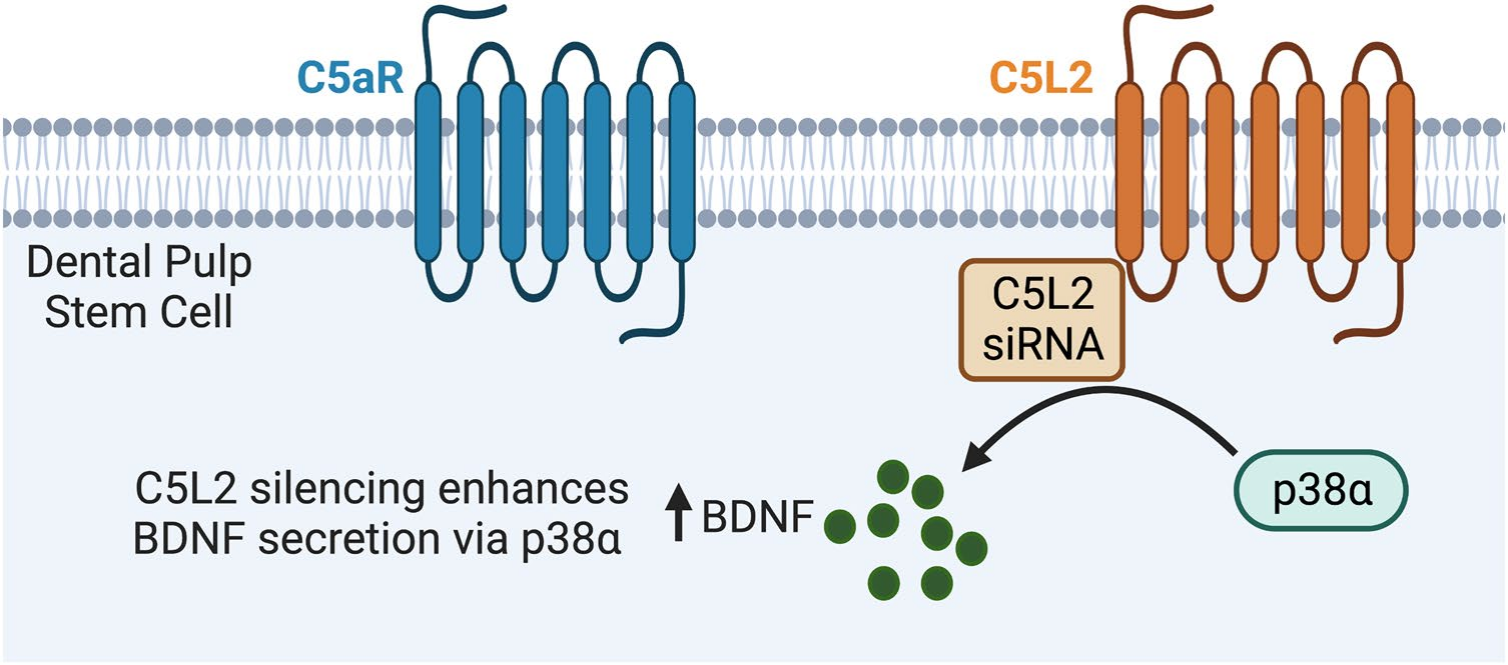
Summarized effects of C5L2 silencing on DPSCs. C5L2 siRNA treatment enhances BDNF secretion via increased phosphorylation of p38α to promote nerve regeneration.

Taken together, we demonstrated for the first time that C5L2 siRNA silencing enhances the BDNF production in DPSCs, with or without LPS. Our data suggest the negative role of C5L2 and propose the mechanistic aspects of C5L2 mediated BDNF secretion via the p38^MAPK^α pathway (Fig. 6). These data could facilitate the future stem cell therapy direction and use of C5L2 as a target molecule considering nerve regeneration and axonal growth.

## Materials and methods

### Chemicals and reagents

Human dental pulp stem cells (DPSCs) were purchased from Lonza, Pharma & Biotech (Cat. # PT-5025). MEM-alpha, PBS, fetal bovine serum, L-glutamine, and Antibiotic–Antimycotic were procured from Gibco™ Fisher Scientific (Waltham, MA, USA). Poly-D-Lysine coated (BioCoat™, 12 mm) round German glass coverslips were purchased from Corning™ Fisher Scientific (Cat. # 354087; Waltham, MA, USA). RIPA buffer was from Cell Signaling Technology (Danvers, MA, USA) and BDNF ELISA kit from R&D System (Minneapolis, MN, USA). Various antibodies were procured: anti-C5L2 was from BioLegend (San Diego, CA, USA), rabbit anti-BDNF from NovusBio (Centennial, CO, USA), mouse anti-STRO-1, mouse anti-p38 and rabbit anti-pp38 from Santa Cruz (Dallas, Texas, USA). Fluorescent secondary antibodies were from Life Technologies (Grand Island, NY, USA). Lipopolysaccharide (LPS) was from Sigma-Aldrich (St. Louis, MO, USA; Cat.# L5418), and chemicals were from Fisher Chemical (Nazareth, PA, USA). siRNA targeting human C5L2, siRNA control and siRNA Reagent System were purchased from Santa Cruz Biotechnology (Dallas, TX, USA).

### Cell culture

Commercially available human DPSCs, which were guaranteed through 10 population doublings, to express CD105, CD166, CD29, CD90, and CD73, and to not express CD34, CD45, and CD133; were further evaluated by immunocytochemistry in cultures with the STRO-1, a stem cell marker. DPSCs were cultured at 37 °C and 5% CO2 in regular/osteogenic media for 72 h in regular growth media (α MEM containing 10% fetal bovine serum (FBS), 1% L-glutamine and antimycotic/ antibiotic), and then treated with p38α inhibitor (SB203580; 10 μM) or LPS (1 μg·mL^−1^). All the experiments were conducted with different sets of DPSCs (between 2nd and 4th passages) 3 times, and cell proliferation was measured by counting the total number of cells.

### Silencing of C5L2 expression by siRNA

Human DPSCs were grown in 6 well plate culture chamber in 2 mL of free-antibiotic medium up to 70% confluence, then transient transfection with siRNAs was performed using the siRNA Reagent System (sc-45064) according to the manufacturer´s protocol. Cells were incubated at 37 °C in a CO_2_ incubator in 1 mL of free-antibiotic and free-serum transfection solution containing a mixture transfection reagent and 40 pmols/mL/well of C5L2 siRNA (sc-105165) or control siRNA, which is a non-targeting siRNA designed as a negative control (sc-37007). After an incubation of 6 h, 1 mL of medium containing 2 times the normal serum and antibiotics concentration was added in each well without removing the transfection mixture. After 24 h, the medium was aspirated and replaced with fresh normal growth medium (DMEM + 4.5 g/L glucose, L-glutamine, sodium, pyruvate + 10% heat-inactivated FBS + 100 μg/mL streptomycin, 100 U/mL penicillin). Assays using siRNA silenced cells were performed within 72 h after the addition of fresh medium.

### Immunocytochemistry

Human DPSCs were seeded at 1 × 10^4^ cells/well on 8-wells glass culture chambers overnight before stimulation with LPS (1 μg/mL). After 24 h, cells were fixed with 4% paraformaldehyde, permeabilized and saturated as previously described ^33^. Then, cells were incubated for 1 h with rabbit anti-BDNF (2.5 μg/mL), and/or rabbit anti-pp38 (2 μg/mL) and/or mouse anti-C5L2 (5 μg/mL) or their respective control isotypes. Finally, cells were incubated for two hours with a mix of Alexa Fluor-594 anti-mouse IgG, Alexa Fluor-488 anti-rabbit IgG (2 μg/mL) and/or DAPI (1 μg/mL). The coverslips were sealed and photographs taken using a Leica DMI6000 B microscope. Fluorescence staining was statistically analyzed by determining the integrated density of each condition using ImageJ 1.49v software. A co-localization analysis was performed using both Colocalization Finder and JACoP plugins on ImageJ software.

### In cell western assay

Human DPSCs were seeded in growth media at 15 × 10^3^ cells/cm^2^ in 96-well plates. At subconfluency, cells were incubated in serum-free medium and/or performed control siRNA or C5L2 siRNA silencing using siRNA reagent systems, and treated with SB203580 and/or LPS (1 μg/mL). Then, cells were immediately fixed with 100% cold methanol (15 min) and saturated with 5% BSA (1.5 h). Cells were incubated overnight at 4 °C with anti-C5L2 (5 μg/mL), anti-BDNF, anti-p38, anti–phospho-p38α (10 g/mL), anti-β-actin or their respective isotype controls. Cells were then washed (0.05% Tween-20/PBS) and incubated with respective IRDye-680RD or IRDye-800RD secondary antibody (1 h). After 5 washes, plates were scanned at 700 and/or 800 nm (Odyssey CLx).

### BDNF quantitative ELISA

Supernatants or cell lysates from DPSCs culture, incubated with various above-mentioned treatments, were collected from cultures (cell lysates were collected using RIPA buffer) after 24 or 48 h and assayed using BDNF ELISA kit according to manufacturer’s protocol (R&D Systems). Briefly, a standard curve was constructed using standards and test samples in duplicate at increasing concentrations and values were normalized accordingly.

### Statistical analysis

The statistical analyses were performed on at least 3 independent experiments with duplicates or triplicates, and statistical significance was determined using one-way analysis of variance (ANOVA) followed by post-hoc Dunnett’s test (SAS 9.4) to compare the different treatments and their respective controls (*p* value of 0.05 or less was considered statistically significant). In addition, the data were also analyzed by Tukey’s test for statistical significance in between the groups. For quantification of immunofluorescence staining intensity, ImageJ 1.49v software was used. Fixed areas of 1 mm × 1 mm or 2 mm × 2 mm were selected to analyze the number or fluorescence intensity of differentiated cells.

## Data Availability

The datasets generated during and/or analyzed during the current study are available from the corresponding author on reasonable request.
